# Expression of Insulin-Like Growth Factor 1 (IGF-1) and Epidermal Growth Factor (EGF) Receptors and the Effect of IGF-1 and EGF on Androgen and Estrogen Release in the Myometrium of Pigs—In Vitro Study

**DOI:** 10.3390/ani10050915

**Published:** 2020-05-25

**Authors:** Ewa Monika Waszkiewicz, Wiktoria Kozlowska, Agata Zmijewska, Anita Franczak

**Affiliations:** Department of Animal Anatomy and Physiology, University of Warmia and Mazury in Olsztyn, Oczapowskiego 1A, 10-719 Olsztyn, Poland; ewa.waszkiewicz@uwm.edu.pl (E.M.W.); kozlowskawiktoria@outlook.com (W.K.); agata.zmijewska@uwm.edu.pl (A.Z.)

**Keywords:** growth factors, IGF-1, EGF, myometrium, pig, steroids, androstenedione, testosterone, estrone, estradiol-17β

## Abstract

**Simple Summary:**

The uterus of pregnant and nonpregnant females produces steroid hormones. The activity of uterine steroidogenesis is altered by the female physiological status, but its regulation is not well understood. This study tested whether growth factors may regulate steroid hormone production in the myometrium. To do so, myometrial slices were isolated from pigs during days 10 or 11, 12 or 13 and 15 or 16 of the estrous cycle and early pregnancy and it was determined whether the tissue possesses potential to respond insulin growth factor 1 (IGF-1) or epidermal growth factor (EGF) and whether they affect androstenedione (A_4_), testosterone (T), estrone (E_1_) and estradiol-17β (E_2_) production in the myometrium. The results showed that myometrial receptivity to IGF-1 and EGF was altered in pregnant and nonpregnant pigs and IGF-1 and EGF affect steroid hormone release in the myometrium. The observed alterations were limited to specific days of the estrous cycle and pregnancy. In conclusion, IGF-1 and EGF may be considered as specific regulators of myometrial steroid hormone production.

**Abstract:**

Porcine myometrium possesses steroidogenic activity but its regulation is not well understood. It was hypothesized that the regulators of myometrial steroidogenesis are insulin-like growth factor 1 (IGF-1) and epidermal growth factor (EGF), which were found to modulate the steroidogenic activity of the endometrium and embryos. Myometrial slices were collected from gravid and nongravid pigs on days 10 or 11, 12 or 13 and 15 or 16 and studied for: (1) the relative abundance of IGF-1R and EGFR mRNA transcripts and proteins, to determine myometrial readiness to response growth factors treatment and (2) the effect of IGF-1 or EGF on the myometrial release of androstenedione (A_4_), testosterone (T), estrone (E_1_) and estradiol-17β (E_2_). The results showed that the relative expression and abundance of IGF-1R and EGFR in the myometrium were altered regarding the female reproductive status. During the estrous cycle, EGF increased myometrial release of A_4_ on days 12–13 and E_2_ on days 15–16. In gravid pigs (days 15–16), IGF-1 and EGF increased the E_1_ release. In conclusion: (1) porcine myometrium possesses the potential to respond to IGF-1 and EGF treatment, (2) EGF significantly increases myometrial A_4_ and E_2_ release in cyclic pigs, while IGF-1 and EGF increase the E_1_ release in gravid pigs.

## 1. Introduction

Insulin-like growth factor 1 (IGF-1) and epidermal growth factor (EGF) are polypeptide mitogens acting through high-affinity tyrosine kinase receptors—IGF-1 receptor (IGF-1R) and EGF receptor (EGFR), respectively [1]. The myometrial expression of IGF-1R and EGFR was found in different species. Specifically, the IGF-1R expression was found in humans [2,3], pigs [4] and rats [5], whereas the expression of EGFR was demonstrated in human [2], mice [6] and goat [7] myometrium. Past studies have shown that the growth factors may act as regulators of gonadal and embryonic steroidogenesis [8,9,10,11]. In mice, EGF was found to reduce ovarian estrogen production [8]. The treatment of oocyte-granulosa cells with EGF resulted in increased progesterone (P_4_), testosterone (T) and estradiol-17β (E_2_) release in vitro [11]. The in vitro studies performed on human granulosa cells collected from preovulatory follicles showed that EGF stimulates P_4_ and E_2_ release while IGF-1 does not alter ovarian steroidogenic activity [12]. In cows, EGF and IGF-1 were found to increase the number of theca cells in vitro, but decreased P_4_ and A_4_ release from the ovarian follicles [13]. In pigs, neither IGF-1 nor EGF affected steroid hormone release in vitro from theca cells [9] while IGF-1 increased estrogen release in elongating conceptuses [10]. It has been established that the porcine myometrium is an important source of steroid hormones [14,15,16,17,18] and only the effect of several factors, i.e., cytokines, adiponectin and orexins on myometrial steroidogenesis, was determined [19,20,21,22,23,24].

It was hypothesized that IGF-1 and EGF affect myometrial steroidogenesis in cyclic and early pregnant pigs and that porcine myometrium possesses the potential to respond to IGF-1 and EGF treatment via IGF-1 and EGF receptors and the action of IGF-1 and EGF in the myometrium might depend on the physiological status of the female. Therefore, this study aimed to determine: (1) the abundance of EGFR and IGF-1R mRNA transcripts and proteins, and (2) the effect of IGF-1 and EGF on A_4_, T, E_1_ and E_2_ release in vitro in porcine myometrium collected during different days of the estrous cycle and early pregnancy.

## 2. Materials and Methods

All experiments were approved by the Animal Ethics Committee, University of Warmia and Mazury in Olsztyn, Poland (ethical approval number 82/2012/DTN). Postpubertal crossbred pigs (Large White × Polish Landrace), aged 10 months, weighing 90 to 110 kg, were used during the estrous cycle or early pregnancy. Gilts were observed for estrus behavior in the presence of an intact boar. The onset of the second estrus was designated as day 0 of the estrous cycle. Gilts assigned to the early pregnancy group were naturally bred twice—on the 1st and the 2nd day of estrus. The animals on days 10 or 11 (*n* = 5), 12 or 13 (*n* = 5) and 15 or 16 (*n* = 5) of the estrous cycle, and on days 10 or 11 (*n* = 5), 12 or 13 (*n* = 5) and 15 or 16 (*n* = 5) of early pregnancy, were slaughtered in a commercial abattoir and the myometrial slices were collected.

**Figure 1:**
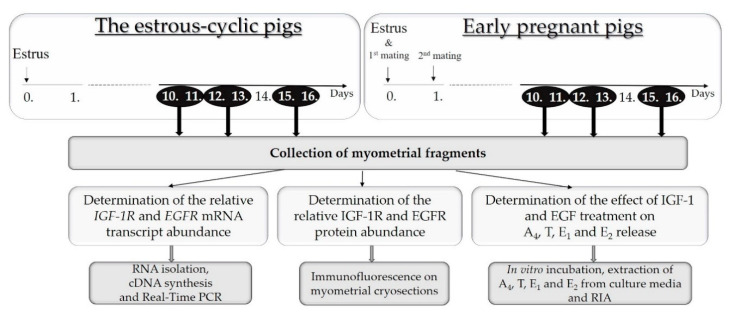
The timeline of the experiment.

The stage of the estrous cycle was estimated and confirmed based on the morphology of ovaries [25]. Pregnancy in mated gilts was confirmed by the presence of embryos after the flushing of each uterine horn with 20 mL of sterile saline. Small sections of the middle part of uterine horns were opened longitudinally on the mesometrial surface and the perimetrium was discarded by careful scraping and slices of the myometrium from the middle part of the uterine horn were then collected with scissors. For gene expression and protein abundance analyses, the myometrial explants were snap-frozen in liquid nitrogen and stored at –80 °C until further analyses. The remaining parts of the uterine horns were placed immediately in ice-cold phosphate-buffered saline (PBS) supplemented with 100 IU/mL penicillin and 100 μg/mL streptomycin and transported to the laboratory on ice for isolation of myometrial slices and in vitro incubation. The timeline of the experiment is presented in Figure 1.

### 2.1. Determination of the Relative Insulin-like Growth Factor 1 Receptor (IGF-1R) and Epidermal Growth Factor Receptor (EGFR) mRNA Transcripts Abundance in the Myometrium

The procedure of transcript abundance analysis was performed according to Bustin et al. [26]. Total RNA was extracted from 30 mg slices of myometrium *n* = 5 for each examined period) using a peqGOLD TriFastTM (Peqlab, Erlangen, Germany), according to the manufacturer’s protocol. The RNA quality and quantity were determined spectrophotometrically (Infinite^®^ 200 PRO, Tecan, Männedorf, Switzerland) and by gel electrophoresis. A 1-µg aliquot of total RNA from each sample was transcribed to cDNA using the QuantiTect Reverse Transcription Kit (Qiagen, Valencia, CA, USA) with a mix of oligonucleotide primers and hexamers, according to the manufacturer’s protocol. Real-time PCR was performed in triplicate for each sample using a 7300 Real-Time PCR System and SYBR^®^ Green PCR Master Mix (both Life Technologies, Grand Island, NY, USA). The conditions of thermal cycling were: (1) initial denaturation, 10 min, 95 °C (one cycle), (2) denaturation, 15 s, 95 °C, primer annealing, 1 min, 60 °C and elongation, 1 min, 72 °C (40 cycles) followed by a dissociation step (one cycle). Specific primers for *IGF-1R* and *EGFR* mRNAs were designed using Primer-BLAST (National Centre for Biotechnology Information, Bethesda, MD, USA) and controlled using Oligo Calc: Oligonucleotide Properties Calculator software [27], Pearl Primer and the mFold web server [28]. The primer’s efficiency was evaluated by creating a standard curve and it was 96.83% for IGF-1R and 96.58% for EGFR. The reference housekeeping genes and the specific primers for their amplification were selected according to Staszkiewicz et al. [29]. The primer sequences are shown in Table 1. The quantitative gene expression was calculated using the *ΔΔCt* method and normalized using the geometrical means of reference gene expression levels: *β-actin* (*ACTB*) and *18S ribosomal RNA* (*RNA 18S*) [30]. Nontemplate controls were used to confirm the amplification specificity for each set of primers. 

### 2.2. Sequence Analysis

After amplification, the putative *IGF-1R* and *EGFR* amplicons were conducted through gel electrophoresis (2.5% agarose) and isolated using GenElute™ Gel Extraction Kit (NA1111, Lot SLBH5865V, Sigma Aldrich, St. Louis, MO, USA). The obtained amplicons were sequenced by Genomed S.A. (Warsaw, Poland). The specificity of amplified amplicons was confirmed by comparison of their sequences with the sequences of *IGF-1R* and *EGFR* deposited in the National Centre for Biotechnology Information (NCBI) database (NM_214007.1 and NM_214172.1, respectively) and calculation of the statistical significance of the match using the Basic Local Alignment Search Tool (BLAST).

### 2.3. Determination of the Relative IGF-1R and EGFR Protein Abundance in the Myometrium

The individual myometrial explants were transferred from –80 °C to –20 °C overnight and then cut at –20 °C in a cryostat (Leica, Weltzar, Germany) on 6-µm-thick slices, put on SuperFrost microscope slides and stored at –20 °C. Before immunofluorescence analysis, the slices were thawed in a humid chamber (room temperature) for 30 min and the individual slices were limited with a PapPen marker (Sigma Aldrich, St. Louis, MO, USA) to avoid the outflow of reagents and buffers. The slices were then fixed with 4% paraformaldehyde (P.P.H STANLAB, Lublin, Poland) for 15 min and then washed in PBS (3 × 5 min). Next, the slices were blocked in PBS with 1% Triton X-100 (MP Biomedicals, Solon, OH, USA) and 10% normal donkey serum (LOT: 2572064A, EMD Millipore, Burlington, MA, USA) at 4 °C for 1 h to avoid nonspecific binding of antibodies. After blocking, the slides were washed in PBS (3 × 5 min) and covered with primary antibodies possessing reactivity with pigs, produced in rabbit and diluted in PBS: anti-IGF-1R (1:200, ab90657, Abcam, Cambridge, Great Britain) for IGF-1R detection and localization and anti-EGFR (1:250, ab32430, Abcam, Cambridge, Great Britain) for EGFR detection and localization. The antibodies dilutions were chosen according to the manufacturer’s protocols. The slices assigned to the negative control group were covered with PBS. The incubation was performed overnight at 4 °C. The next day, the slices were washed in PBS (3 × 5 min) and covered with donkey anti-rabbit secondary antibodies Alexa Fluor 555 suspended in PBS (1:1500, A31572, LOT 1671993, Life Technologies, Eugene, OR, USA). The labeling of secondary antibodies was performed in darkness to prevent photobleaching, at 4 °C for 1 h. Next, the slices were washed in PBS (3 × 5 min) and mounted with Fluoroshield with DAPI (F6057, LOT: SLBH0683, Sigma Aldrich, St. Louis, MO, USA). Mounted slides were dried at 4 °C and then analyzed using an epifluorescent BX 51 microscope (Olympus, Tokyo, Japan). The images were archived with a digital camera type DP72 (Olympus, Japan), under 400-fold magnification. The data were collected out of 20 fields of each myometrial slice. The intensity of the immunofluorescence signal was calculated with Cell^F software (Olympus, Tokyo, Japan) and normalized with the intensity of the immunofluorescence signal of negative controls for each group.

### 2.4. Incubation of Myometrial Slices

Individual myometrial slices (200–210 mg, 3-mm-thick, two slices per each treatment) and dose, i.e., control, IGF-1 (10 or 100 ng/mL) and EGF (10 or 100 ng/mL) from each animal (*n* = 5 per each period of the estrous cycle or pregnancy, i.e., days 10–11, 12–13 and 15–16), were washed twice with PBS supplemented with 100 IU/mL penicillin and 100 μg/mL streptomycin and then placed in culture vials containing 2 mL of Medium 199 (Sigma Aldrich, St. Louis, MO, USA) supplemented with 0.1% bovine serum albumin (BSA) fraction V (Sigma Aldrich, St. Louis, MO, USA) and 20 μg gentamycin (Sigma Aldrich, St. Louis, MO, USA). First, the slices were preincubated in fresh control medium in a shaking water bath at 37 °C in an atmosphere of 95% O_2_ and 5% CO_2_ for 18 h according to the previous studies [18,19,23] and incubated for 6 h in fresh control medium or fresh medium supplemented with IGF-1 (The National Hormone and Peptide Program (NHPP), Dr. A.F. Parlow; 10 ng/mL or 100 ng/mL) or EGF (E4127, Sigma Aldrich, St. Louis, MO, USA 10 ng/mL or 100 ng/mL). The control myometrial slices, i.e., incubated in the control medium without IGF-1 or EGF treatment were prepared for each animal. The duration of in vitro incubation was selected based on the results of the author’s previous studies concerning the basal and treatment-stimulated steroidogenesis in the myometrium [16,19]. The doses of growth factors were selected based on earlier studies: IGF-1 [31] and EGF [32]. After incubation, the culture vials were placed in an ice bath and the culture medium was collected and frozen at –20 °C until a determination of A_4_, T, E_1_ and E_2_ concentration with radioimmunoassay (RIA).

### 2.5. Determination of A_4_, T, E_1_ and E_2_ Concentration in the Media

Concentrations of A_4_, T, E_1,_ and E_2_ in media were determined with RIA, according to the standard protocol used in the previous studies in the author’s laboratory and antibodies described by Szafranska et al. [33]. The efficiency of extraction for A_4_, T, E_1_ and E_2_ was 85.0% ± 0.1%, 87.1% ± 0.1%, 86.3% ± 0.1% and 84.2% ± 0.1%, respectively. The assay sensitivity for A_4_, T, E_1,_ and E_2_ was 1 pg/mL. The intra- and interassay coefficients of variation for A_4_, T, E_1_ and E_2_ were 0.96%, 1.02%, 0.85%, 1.28%, respectively, and 5.84%, 4.86%, 8.60% and 9.51%, respectively.

### 2.6. Statistical Analysis

All of the data are presented as mean and SEM. Statistical analyses were performed using Statistica v. 13.1 software (Dell, Round Rock, TX, USA). Statistically significant differences were considered with *p* ≤ 0.05. Data obtained with Real-Time PCR were conducted through *ΔΔCt* analysis and 2*^-ΔΔCt^* values were used for statistical calculations. The data obtained with immunofluorescence analysis were normalized to negative controls and used without mathematical transformation. The data obtained with RIA analysis for statistical calculations were log-transformed. The relative abundance of *IGF-1R* and *EGFR* mRNA transcripts and encoded proteins abundance was analyzed with multiway ANOVA followed by Fisher’s LSD post hoc test, using a physiological status (the estrous cycle or pregnancy) and day of the estrous cycle or pregnancy (10–11, 12–13 or 15–16) as independent variables. To define the main factors affecting steroid hormones release, a multiway ANOVA, using independent variables was performed, as indicated above. To determine the effect of IGF-1 or EGF treatment within the specific day of the estrous cycle or pregnancy, a one-way ANOVA followed by Dunnett’s post hoc test (comparison to the control, i.e., slices of myometrium collected from pigs within the specific day of the estrous cycle or pregnancy and not treated with IGF-1 or EGF) was performed.

## 3. Results

### 3.1. The Relative Abundance of EGFR and IGF-IR mRNA Transcripts and Proteins in the Myometrium

#### 3.1.1. The Main Effects and Interactions Affecting the Relative Abundance of EGFR and IGF-1R mRNA Transcripts and Proteins

The day of the estrous cycle or pregnancy effect was found to have a statistically significant impact on the relative *EGFR* mRNA transcript and protein abundances. The interaction among physiological status and day of the estrous cycle or pregnancy significantly influenced the EGFR protein abundance. The details concerning the *F* statistic value and *p*-value are presented in Table 2.

#### 3.1.2. The Relative IGF-1R and EGFR mRNA Transcript Abundances

**Figure 2:**
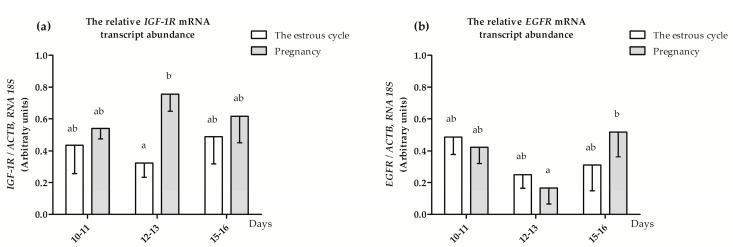
The relative abundance of (**a**) *IGF-1R* and (**b**) EGFR mRNA transcripts in the myometrium collected from pigs during the estrous cycle (days 10–11, 12–13 and 15–16) and corresponding days of early pregnancy (multiway ANOVA, Fisher’s LSD post hoc test). Statistically significant differences were considered at *p* ≤ 0.05 and indicated as lower-case letters (a,b) above the bars.

The relative *IGF-1R* mRNA transcript abundance was significantly lower (*p* ≤ 0.05) during days 12–13 of the estrous cycle compared to days 12–13 of early pregnancy and did not differ (*p* > 0.05) among the other days of the estrous cycle and pregnancy (Figure 2a). The relative *EGFR* mRNA transcript abundance was significantly greater (*p* ≤ 0.05) in myometrial fragments collected during days 15–16 of pregnancy compared to days 12–13 of pregnancy and did not differ (*p* > 0.05) during the remaining days of the estrous cycle and pregnancy (Figure 2b).

#### 3.1.3. The IGF-1R and EGFR Protein Location and Abundance

**Figure 3:**
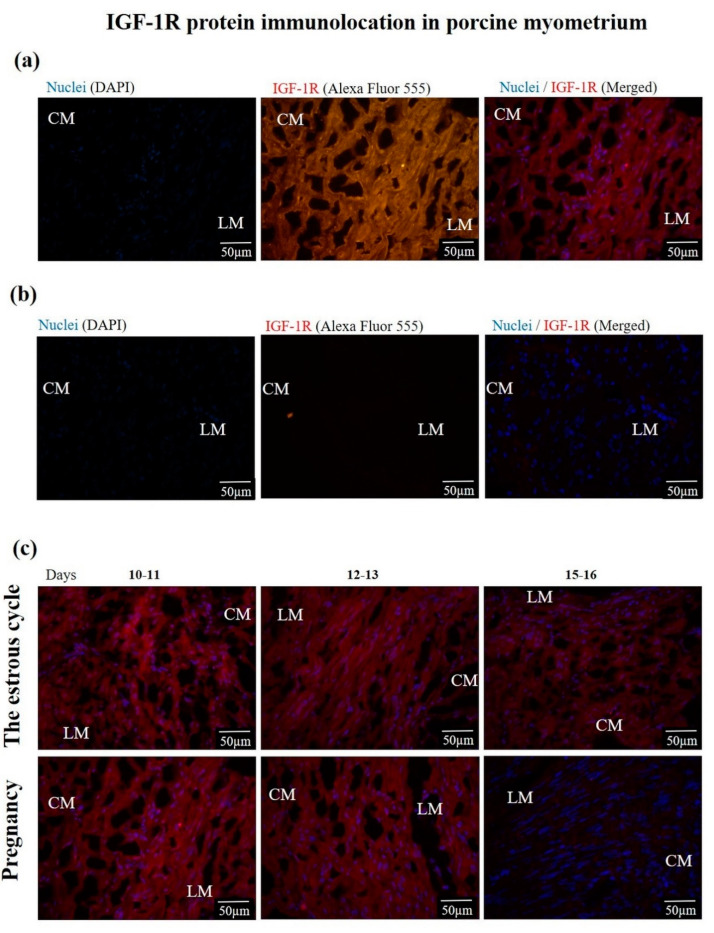
The representative photographs showing (**a**) IGF-1R immunostaining, (**b**) negative control, and (**c**) the myometrial location of IGF-1R during days 10–11, 12–13 and 15–16 of the estrous cycle and days 10–11, 12–13 and 15–16 of pregnancy. Magnification 400×. CM—circular muscle; LM—longitudinal muscle. The nuclei are stained with DAPI (**blue**) and IGF-1R sites are stained with Alexa Fluor 555 (**red**).

**Figure 4:**
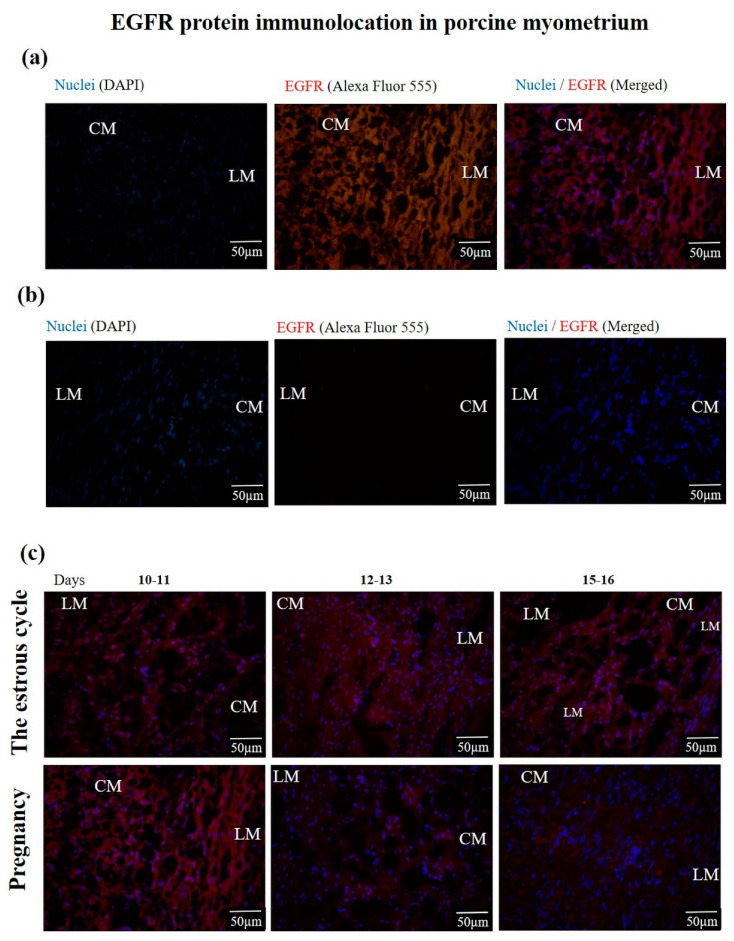
The representative photographs showing (**a**) EGFR immunostaining, (**b**) negative control, and (**c**) the myometrial location of EGFR during days 10–11, 12–13 and 15–16 of the estrous cycle and days 10–11, 12–13 and 15–16 of pregnancy. Magnification 400×. CM—circular muscle; LM—longitudinal muscle. The nuclei are stained with DAPI (**blue**) and EGFR sites are stained with Alexa Fluor 555 (**red**).

**Figure 5:**
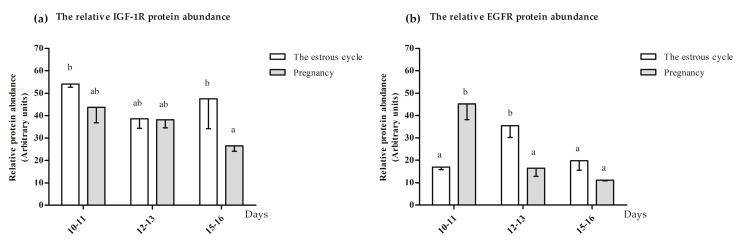
The relative abundance of (**a**) IGF-1R and (**b**) EGFR proteins in the myometrium collected from pigs during the estrous cycle (days 10–11, 12–13 and 15–16) and corresponding days of early pregnancy (multiway ANOVA, Fisher’s LSD post hoc test). Statistically significant differences were considered at *p* ≤ 0.05 and indicated as lower-case letters (a,b) above the bars.

IGF-1R and EGFR proteins were found in the circular and longitudinal layers of the myometrium. The representative photographs visualizing the location of IGF-1R and EGFR proteins are presented in Figure 3 and Figure 4. The IGF-1R protein abundance was significantly increased (*p* ≤ 0.05) on days 10–11 and 15–16 of the estrous cycle compared to days 15–16 of pregnancy and was not altered (*p* > 0.05) compared to the other days of the estrous cycle and early pregnancy (Figure 5a). The EGFR protein abundance was the highest (*p* ≤ 0.05) on days 10–11 of pregnancy and 12–13 of the estrous cycle compared to other days of the estrous cycle and early pregnancy (Figure 5b).

### 3.2. The Effect of IGF-1 and EGF on the A_4_, T, E_1_ and E_2_ Release from the Myometrium

#### 3.2.1. The Main Effects and Interactions Affecting Myometrial Steroid Hormone Release 

The main effects and interactions observed among factors affecting steroid hormones release, i.e., treatment, female physiological status and days studied of the estrous cycle or pregnancy, are presented in Table 3. The A_4_ release was affected by treatment (*p* = 0.037423) and day of the estrous cycle or pregnancy (*p* = 0.000120) and the interaction between physiological status and day of the estrous cycle or pregnancy (*p* = 0.000000). The T release was altered only when physiological status and day of the estrous cycle or pregnancy were interacting (*p* = 0.000000). The E_1_ release was affected by treatment (*p* = 0.009750) and physiological status (*p* = 0.03931). For E_2_ release, none of the main effects were evaluated.

#### 3.2.2. Androstenedione (A_4_) and Testosterone (T) Release

**Figure 6:**
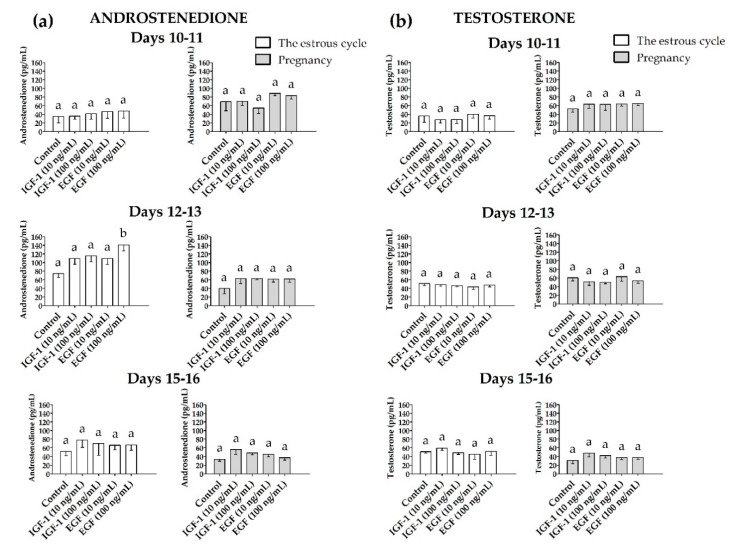
The concentration of (**a**) androstenedione and (**b**) testosterone in media collected after in vitro incubation of myometrial slices treated with IGF-1 (10 and 100 ng/mL) or EGF (10 and 100 ng/mL, one-way ANOVA, Dunnett’s post hoc test). Statistically significant differences were considered at *p* ≤ 0.05 and indicated as lower-case letters (a,b) above the bars.

The A_4_ release from the myometrial slices was significantly increased (*p* ≤ 0.05) only on days 12–13 of the estrous cycle in the presence of EGF (100 ng/mL) when compared to the control (Figure 6a). On days 10–11 and 15–16 of the estrous cycle and during all days of pregnancy, neither EGF nor IGF-1 affected the A_4_ release (*p* > 0.05). IGF-1 and EGF did not alter (*p* > 0.05) T release from myometrial slices compared to the control in either gravid or nongravid pigs (Figure 6b).

#### 3.2.3. Estrone (E_1_) and Estradiol-17β (E_2_) Release

**Figure 7:**
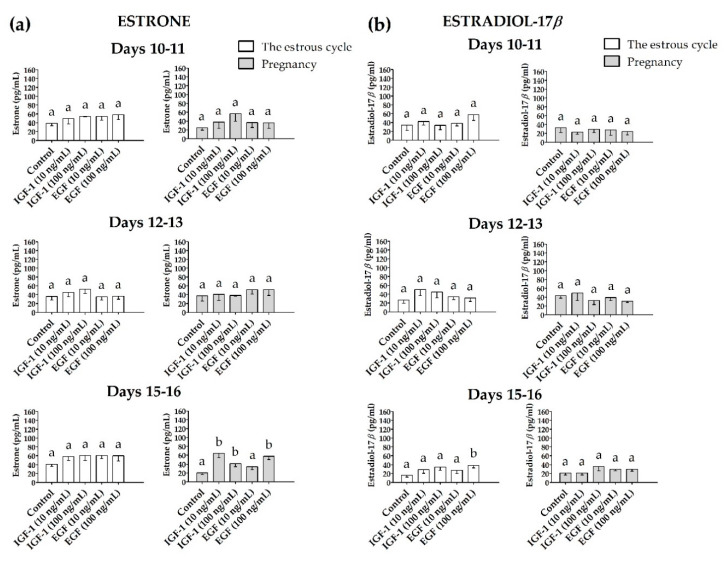
The concentration of (**a**) estrone and (**b**) estradiol-17β in media collected after in vitro incubation of myometrial slices treated with IGF-1 (10 and 100 ng/mL) or EGF (10 and 100 ng/mL, one-way ANOVA, Dunnett’s post hoc test). Statistically significant differences were considered at *p* ≤ 0.05 and indicated as lower-case letters (a,b) above the bars.

The myometrial E_1_ release was increased (*p* ≤ 0.05) only on days 15–16 of pregnancy in the presence of IGF-1 (10 and 100 ng/mL) and EGF (100 ng/mL) when compared to the control and did not alter (*p* > 0.05) in the other days studied of the estrous cycle and pregnancy (Figure 7a). The myometrial release of E_2_ was increased (*p* ≤ 0.05) only on days 15–16 of the estrous cycle in the presence of EGF (100 ng/mL, Figure 7b).

## 4. Discussion

The current study enriches the knowledge concerning the regulation of myometrial steroidogenesis in pigs. First, this study confirmed that the relative abundance of IGF-1R and EGFR mRNA transcripts and proteins in the myometrium is altered by the female reproductive status. This finding suggests the unequal readiness of porcine myometrium to synthesize IGF-1R and EGFR and respond to IGF-1 and EGF in the following days of the estrous cycle and early pregnancy. Second, this study found that myometrial slices collected during the peri-implantation period and treated in vitro with IGF-1 release relatively greater amounts of E_1_. Moreover, the myometrium treated in vitro with EGF used at the dose of 100 ng/mL release relatively greater amounts of A_4_ when collected during the mid-luteal phase of the estrous cycle, E_2_ when collected during luteolysis, and E_1_ when collected during the peri-implantation period. These notions indicate that in the presence of IGF-1 and EGF, myometrial steroidogenic activity is altered in vitro and the final results differ regarding the physiological status of the female.

Selected days of the estrous cycle and pregnancy (i.e., days 10–11, 12–13 and 15–16) are important for the success of pig reproduction. In gravid gilts, on days 10–11 of pregnancy, embryos enter the uterine horns, start to migrate and enlarge. On days 12–13 of pregnancy, the enlargement of embryos is continued and accelerated and the embryos start their apposition. Finally, on days 15-16 of pregnancy, embryos start the implantation process [34,35,36,37]. Days 15–16 of pregnancy are accompanied by the estrogen-controlled prostaglandin F_2_α (PGF_2_α) retrograde transfer from the utero-ovarian vein, which is involved in the protection of corpora lutea (CLs) from luteal regression [37,38,39]. In nonpregnant gilts, days 10–11 and 12–13 of the estrous cycle are recognized as increasing activity of CLs, while days 15–16 the estrous cycle are the period when CLs are regressed and new waves of ovarian follicles are recruited [37,38,39]. Thus, to compare the in vitro effect of IGF-1 and EGF treatment in cyclic and early pregnant pig myometrium, the respective days of the estrous cycle and pregnancy were selected.

The presence of EGF-EGFR in the porcine uterus was previously demonstrated only in the endometrium for rev. [40] and uterine arteries [41] while a comparative transcriptomic study revealed the abundance of *EGF* mRNA transcript in porcine myometrium [42]. Interestingly, during the mid-luteal phase of the estrous cycle, i.e., on days 12–13, the expression of *EGF* mRNA was decreased compared to the respective days of pregnancy [42]. The current study demonstrates that myometrial slices collected during the mid-luteal phase of the estrous cycle and exposed in vitro to EGF treatment release greater amounts of A_4_. It is worth noting that during the estrous cycle, the basal myometrial release of A_4_ was greater on days 12–13 than on days 10–11 and 15–16 of the estrous cycle [18]. In the current study, it was documented that EGF may be recognized as a potential enhancer of myometrial A_4_ production during the mid-luteal phase of the estrous cycle, but only when used at a higher dose (100 ng/mL). The observed phenomenon can be specific, since the A_4_ release was affected by the treatment, day of the estrous cycle or pregnancy and by the interaction between physiological status and days of the estrous cycle or pregnancy. It was also indicated that specifically during days 12–13 of the estrous cycle, myometrial production of A_4_ in response to EGF treatment is accompanied by an increased abundance of EGFR protein, but not mRNA in the tissue. Interestingly, on days 15–16 of the estrous cycle and early pregnancy, EGF increased the secretion of myometrial estrogens, but the abundance of EGFR protein in the tissue was lowered when compared to tissue during the mid-luteal phase of the estrous cycle, despite the unaltered concentration of *EGFR* mRNA. It is visible that the myometrial expression of EGFR mRNA does not correspond with the abundance of the encoded protein and it is hard to find a strict relation between myometrial EGFR and the effect of EGF on steroid hormone production in this tissue. These alterations may result from transcriptional and post-transcriptional regulations, RNA stability and processing as well as translational and post-translational processes [43,44]. Low protein concentrations accompanying high gene expression may be caused by the action of RNA interference [45]. High protein concentration may suppress mRNA expression and a high level of gene expression may diminish the post-translational modifications and affect the encoded protein level [46]. Therefore, the observed no differences in IGF-1R and EGFR mRNAs during the experimental periods accompanying significant differences in IGF-1R protein abundance between days 10–11 and 15–16 of the estrous cycle, and EGFR protein abundance during the estrous cycle and pregnancy is not a surprising phenomenon.

The results from the present study provided evidence that *IGF-1R* mRNA transcript abundance is increased only in pigs during days 12–13 of pregnancy when compared to respective days of the estrous cycle and does not alter when compared to other studied days in pregnant and estrous-cyclic pigs. On the protein level, IGF-1R was increased in cyclic pigs on days 10–11 and 15–16 only when compared to days 15–16 of pregnancy. Thus, myometrial sensitivity to IGF-1 appears to be equal during days 10–11 and 12–13 of the estrous cycle and early pregnancy but is markedly lesser during the peri-implantation period as compared with pigs during luteolysis. Interestingly, in pregnant pigs, the uterine content of IGF-1 is high at the time accompanying rapid elongation of porcine conceptuses and then decreases after day 13th of pregnancy [47]. Moreover, IGF-1 was found to function as an enhancer of the aromatase p450 activity in porcine conceptuses [10]. It is possible that observed in the current study lesser abundance of myometrial IGF-1R could indirectly prevent oversecretion of E_2_ from the myometrium during the peri-implantation period. It is worth highlighting that the effect of IGF-1 and EGF on E_2_ release during the fetal peri-implantation period was not observed. Interestingly, during the fetal peri-implantation period (days 15–16 of pregnancy) *EGFR* mRNA transcript abundance was markedly greater when compared to the time of maternal recognition of pregnancy (days 12–13 of pregnancy), whereas EGFR protein abundance did not alter. When there is an increased abundance of *EGFR* mRNA, as observed during the periconceptional period, the myometrium should possess greater potential to synthesize EGFR protein, but, possibly due to transcriptional and/or post-transcriptional modifications [45] the protein amount is not greater. On the other hand, as the high protein concentration may suppress mRNA expression [46], the increased abundance of EGFR protein on days 10–11 of pregnancy could lead to inhibition of *EGFR* mRNA transcript abundance as observed on days 12–13 of pregnancy.

It is worth noting that A_4_ in pigs is the most potent androgen [48]. The EGF and A_4_ were found to induce anabolic processes in the target tissue and regulate morphogenesis, cellular proliferation, hyperplasia and aging [49]. It is worth noting that the mid-luteal phase of the estrous cycle in pigs is accompanied by increased uterine weight (approximately 40%–60%) compared to the early luteal phase of the estrous cycle [39]. This suggests that these morphological changes occurring in the uterus may be mediated by EGF directly, i.e., by the regulation of anabolic processes in the tissue, as well as indirectly, i.e., through the induction of A_4_ synthesis and release from the myometrium. The current study demonstrated that EGF stimulates also myometrial E_2_ production during luteolysis, but only when EGF was used at higher doses. The past in vitro study documented that myometrial E_2_ production can be increased by interleukin 6 (IL6), interleukin 1β (IL1β) and tumor necrosis factor α (TNFα), but only during the peri-implantation period, not during luteolysis [23]. Therefore, it appears that EGF, used at higher doses, is the only one acting as an enhancer of myometrial E_2_ production during luteolysis. It is worth noting that myometrial production of E_2_ during luteal regression is about 59% of total uterine E_2_ production and the myometrium appears to be a preferential source of uterine E_2_ [15]. Respecting the mechanism of estrogen action [50], it cannot be excluded that myometrial E_2_, released in response to EGF, may trigger transcriptional activation of genes expressed in the uterus during luteolysis [51,52,53].

Interestingly, EGF was found to upstream the expression of several genes that were found to be differentially expressed in gilts during the peri-implantation period vs. luteolysis [53]. The current study determined, for the first time, that both EGF (100 ng/mL) and IGF-1 (10 and 100 ng/mL) increased myometrial production of E_1_ on days 15–16 of pregnancy. Estrone is known as an estrogen with reduced potency but, via the activity of 17β-hydroxysteroid dehydrogenase (17β-HSD), it may be converted to a more potent E_2_ [54]_._ The presence of 17β-HSD was documented in porcine endometrium [55], but it cannot be neglected that the phenomenon of E_1_ to E_2_ conversion may also occur in the myometrium. Thus, EGF (100 ng/mL) and IGF-1 (10 and 100 ng/mL) by stimulating E_1_ release from the myometrium may indirectly cause a greater myometrial production of E_2_ during the peri-implantation period. According to a two-signal switch hypothesis, including the role of E_2_ during early pregnancy in the appearance of prostaglandin F_2_α (PGF_2_α) retrograde transfer and the creation of the increased ratio of PGE_2_ to PGF_2_α [56], myometrial E_2_ in gravid pigs may supplement the “rescue switch” from embryo signals to protect CLs.

Interestingly, myometrial production of E_1_ stimulated by IGF-1 during the peri-implantation period occurs despite the decreased concentration of IGF-1R observed in the tissue. This effect may result from the observed decreasing uterine content of IGF binding proteins (IGFBPs), which are responsible for preventing receptor–ligand docking [57]. It was found that IGFBPs are synthesized by porcine myometrium [58], although the pattern of IGFBPs synthesis and release has not yet been studied.

Previous studies showed that the myometrial production of E_1_ is regulated by a variety of factors. It was demonstrated that orexin A (OXA) and orexin B (OXB) increase the myometrial production of E_1_ in selected studied days of early pregnancy and the estrous cycle in pigs [20,22]. Other studies showed that the regulation of myometrial E_1_ production may also be physiological-status-related. For example, increased myometrial E_1_ production was observed in response to IL6 during the mid-luteal phase of the estrous cycle (days 12–13) and luteolysis (days 15–16 of the estrous cycle), while in pregnant females the myometrial production of E_1_ was increased in the response of the tissue to IL6 and IL1β action specifically during maternal recognition of pregnancy period, i.e., on days 12–13 of pregnancy [19]. In this study, it was demonstrated that in vitro treatment with IGF-1 (10 and 100 ng/mL) and EGF (100 ng/mL) increased myometrial E_1_ production only during the peri-implantation period. Hence, it appears that IGF-1 and EGF, similarly to cytokines, might be recognized as physiological-status-specific enhancers of myometrial E_1_ production.

## 5. Conclusions

In conclusion: (1) in porcine myometrium, EGF, used at a dose of 100 ng/mL, significantly increases A_4_ during the mid-luteal phase of the estrous cycle and E_2_ release during luteolysis; (2) during the peri-implantation period, IGF-1 and EGF increase myometrial E_1_ secretion; (3) EGF, by increasing myometrial A_4_ production, may provide a substrate for estrogen synthesis and affect myometrial morphogenesis during the mid-luteal phase of the estrous cycle; (4) both IGF-1 and EGF contribute to increasing the intrauterine content of estrogens of a myometrial source during luteolysis and the peri-implantation period.

## Figures and Tables

**Table 1:** Used primer sequences.

Gene Symbol	Primer Sequence (5’→3’)	Gene Bank Accession No.	Annealing (°C)	Amplicon Length (bp)
*IGF-1R*	F: TTTGTGCCCAGACCTGAACG R: GTAAAAGGCCGGAGGTTGGA	NM_214172.1	60	200
*EGFR*	F: TTCCGTCCGAAACAATCGG R: TTCCCAGTGAGGCACAGAG	NM_214007.1	60	103
*ACTB*	F: GGAGATCGTGCGGGACATCAAG R: GGCGTAGAGGTCCTTCCTGATG	U07786.1	60	268
*RNA 18S*	F: ACTGAGGCCATGATTAAG R: GCTATCAATCTGTCAATCC	AF102857.1	60	521

**Table 2:** *Insulin-like Growth Factor 1 Receptor* (*IGF-IR*) and *Epidermal Growth Factor Receptor* (*EGFR*) mRNAs expression and EGFR and IGF-IR protein abundance—the main effects and the interactions observed among factors (multiway ANOVA).

Effect	*IGF-1R* mRNA		*EGFR* mRNA	
	*F*	*p*-Value	*F*	*p*-Value
Physiological status ^1^	2.44879	0.136039	0.09807	0.757970
Day of the estrous cycle or pregnancy ^2^	0.45379	0.642693	3.71779	0.045775
Physiological status × Day of the estrous cycle or pregnancy	0.38678	0.685068	0.13592	0.873849
**Effect**	***IGF-1R* Protein**		***EGFR* Protein**	
	***F***	***p*-Value**	***F***	***p*-Value**
Physiological status ^1^	3.8520	0.065331	0.0022	0.962905
Day of the estrous cycle or pregnancy ^2^	1.9167	0.175939	4.2889	0.029976
Physiological status × Day of the estrous cycle or pregnancy	1.1946	0.325719	12.9711	0.000325

^1^ The estrous cycle or pregnancy; ^2^ Days 10–11, 12–13 or 15–16.

**Table 3:** Myometrial A_4_, T, E_1_ and E_2_ release—the main effects and the interactions observed among factors (multiway ANOVA).

Factors	A_4_		T		E_1_		E_2_	
	*F*	*p*-Value	*F*	*p*-Value	*F*	*p*-Value	*F*	*p*-Value
Treatment ^1^	2.659	0.037423	0.36	0.836151	3.529	0.009750	1.111	0.355716
Physiological status ^2^	1.378	0.243377	5.85	0.017356	8.716	0.003931	1.627	0.205110
Days of the estrous cycle or pregnancy ^3^	9.939	0.000120	2.39	0.096971	1.312	0.273793	2.081	0.130233
Treatment × Physiological status	0.169	0.953780	0.21	0.931675	0.481	0.749270	1.470	0.216833
Treatment × Days of the estrous cycle or pregnancy	0.743	0.653462	0.90	0.517208	0.723	0.670727	1.212	0.299688
Physiological status × Days of the estrous cycle or pregnancy	17.850	0.000000	18.44	0.000000	2.838	0.063284	2.402	0.095745
Treatment × Physiological status × Days of the estrous cycle or pregnancy	0.167	0.994718	0.44	0.895146	0.845	0.565218	0.381	0.928360

^1^ IGF-1 or EGF (10 ng/mL or 100 ng/mL); ^2^ The estrous cycle or pregnancy; ^3^ Days 10–11, 12–13 or 15–16.

## References

[1] Lemmon M.A., Schlessinger J. (2010). Cell signaling by receptor tyrosine kinases. Cell.

[2] Konopka B., Skasko E., Kluska A., Goluda M., Janiec-Jankowska A., Paszko Z., Ujec M. (1998). Changes in the concentrations of receptors of insulin-like growth factor-I, epithelial growth factor, oestrogens and progestagens in adenomyosis foci, endometrium and myometrium of women during menstrual cycle. Eur. J. Gynaecol. Oncol..

[3] Tang X.-M., Rossi M.J., Masterson B.J., Chegini N. (1994). Insulin-Like Growth Factor I (IGF-I), IGF-I Receptors, and IGF Binding Proteins 1–4 in Human Uterine Tissue: Tissue Localization and IGF-I Action in Endometrial Stromal and Myometrial Smooth Muscle Cells in Vitro. Biol. Reprod..

[4] Hofig A., Michel F.J., Simmen F.A., Simmen R.C.M. (1991). Constitutive Expression of Uterine Receptors for Insulin-Like Growth Factor-I during the Peri-Implantation Period in the Pig1. Biol. Reprod..

[5] Shynlova O., Tsui P., Dorogin A., Langille B.L., Lye S.J. (2007). Insulin-like Growth Factors and Their Binding Proteins Define Specific Phases of Myometrial Differentiation During Pregnancy in the Rat. Biol. Reprod..

[6] Das S.K., Tsukamura H., Paria B.C., Andrews G.K., Dey S.K. (1994). Differential expression of epidermal growth factor receptor (EGF-R) gene and regulation of EGF-R bioactivity by progesterone and estrogen in the adult mouse uterus. Endocrinology.

[7] Tamada H., Yoh C., Inaba T., Takano H., Kawate N., Sawada T. (2000). Epidermal growth factor (EGF) in the goat uterus: Immunohistochemical localization of EGF and EGF receptor and effect of EGF on uterine activity in vivo. Theriogenology.

[8] Hsueh A.J.W., Welsh T.H., Jones P.B.C. (1981). Inhibition of ovarian and testicular steroiodogenesis by epidermal growth factor. Endocrinology.

[9] Caubo B., Devinna R.S., Tonetta S.A. (1989). Regulation of steroidogenesis in cultured porcine theca cells by growth factors. Endocrinology.

[10] Green M.L., Simmen R.C.M., Simmen F.A. (1995). Developmental regulation of steroidogenic enzyme gene expression in the periimplantation porcine conceptus: A paracrine role for insulin-like growth factor-I. Endocrinology.

[11] Jamnongjit M., Gill A., Hammes S.R. (2005). Epidermal growth factor receptor signaling is required for normal ovarian steroidogenesis and oocyte maturation. Proc. Natl. Acad. Sci. USA.

[12] Gaetje R. (1994). IGF-I and EGF influence on steroid secretion and morphology of human granulosa cells of IVF-cycles and natural cycles in vitro. Clin. Exp. Obstet. Gynecol..

[13] Spicer L.J., Stewart R.E. (1996). Interactions among basic fibroblast growth factor, epidermal growth factor, insulin, and insulin-like growth factor-I (IGF-I) on cell numbers and steroidogenesis of bovine thecal cells: Role of IGF-I receptors. Biol. Reprod..

[14] Franczak A. (2008). Endometrial and myometrial secretion of androgens and estrone during early pregnancy and luteolysis in pigs. Reprod. Biol..

[15] Franczak A., Kotwica G. (2008). Secretion of estradiol-17β by porcine endometrium and myometrium during early pregnancy and luteolysis. Theriogenology.

[16] Franczak A., Kotwica G. (2010). Androgens and estradiol-17β production by porcine uterine cells: In vitro study. Theriogenology.

[17] Franczak A., Zmijewska A., Kurowicka B., Wojciechowicz B., Kotwica G. (2010). Interleukin 1β-induced synthesis and secretion of prostaglandin E2 in the porcine uterus during various periods of pregnancy and the estrous cycle. J. Physiol. Pharmacol..

[18] Wojciechowicz B., Kotwica G., Kolakowska J., Franczak A. (2013). The Activity and Localization of 3β-hydroxysteroid Dehydrogenase/Δ 5-Δ 4 Isomerase and Release of Androstenedione and Progesterone by Uterine Tissues During Early Pregnancy and the Estrous Cycle in Pigs. J. Reprod. Dev..

[19] Franczak A., Wojciechowicz B., Katwica G. (2013). Novel aspects of cytokine action in porcine uterus—endometrial and myometrial production of estrone (E1) in the presence of interleukin 1β (Il1β), interleukin 6 (Il6) and tumor necrosis factor (TNFα)—in vitro study. Folia Biol..

[20] Kiezun M., Smolinska N., Dobrzyn K., Szeszko K., Rytelewska E., Kaminski T. (2017). The effect of orexin A on CYP17A1 and CYP19A3 expression and on oestradiol, oestrone and testosterone secretion in the porcine uterus during early pregnancy and the oestrous cycle. Theriogenology.

[21] Kisielewska K., Rytelewska E., Gudelska M., Kiezun M., Dobrzyn K., Szeszko K., Bors K., Wyrebek J., Kaminski T., Smolinska N. (2019). The effect of orexin B on steroidogenic acute regulatory protein, P450 side-chain cleavage enzyme, and 3β-hydroxysteroid dehydrogenase gene expression, and progesterone and androstenedione secretion by the porcine uterus during early pregnancy and the estrous cycle. J. Anim. Sci..

[22] Kaminski T., Smolinska N., Kiezun M., Dobrzyn K., Szeszko K., Maleszka A. (2018). Effect of orexin B on CYP17A1 and CYP19A3 expression and oestradiol, oestrone and testosterone secretion in the porcine uterus during early pregnancy and the oestrous cycle. Animal.

[23] Franczak A., Wojciechowicz B., Kolakowska J., Kotwica G. (2014). The effect of interleukin-1β, interleukin-6, and tumor necrosis factor-α on estradiol-17β release in the myometrium: The invitro study on the pig model. Theriogenology.

[24] Smolinska N., Dobrzyn K., Kiezun M., Szeszko K., Maleszka A., Kaminski T. (2016). Effect of adiponectin on the steroidogenic acute regulatory protein, P450 side chain cleavage enzyme and 3β-hydroxysteroid dehydrogenase gene expression, progesterone and androstenedione production by the porcine uterus during early pregnancy. J. Physiol. Pharmacol..

[25] Akins E.L., Morrissette M.C. (1968). Gross ovarian changes during estrous cycle of swine. Am. J. Vet. Res..

[26] Bustin S.A., Benes V., Garson J.A., Hellemans J., Huggett J., Kubista M., Mueller R., Nolan T., Pfaffl M.W., Shipley G.L. (2009). The MIQE guidelines: Minimum information for publication of quantitative real-time PCR experiments. Clin. Chem..

[27] Kibbe W.A. (2007). OligoCalc: An online oligonucleotide properties calculator. Nucleic Acids Res..

[28] Zuker M. (2003). Mfold web server for nucleic acid folding and hybridization prediction. Nucleic Acids Res..

[29] Staszkiewicz J., Skowronski M.T., Siawrys G., Kaminski T., Krazinski B.E., Plonka K.J., Wylot B., Przala J., Okrasa S. (2007). Expression of proopiomelanocortin, proenkephalin and prodynorphin genes in porcine luteal cells. Acta Vet. Hung..

[30] Livak K.J., Schmittgen T.D. (2001). Analysis of relative gene expression data using real-time quantitative PCR and the 2-ΔΔCT method. Methods.

[31] Kaczmarek M.M., Blitek A., Schams D., Ziecik A.J. (2008). The effect of insulin-like growth factor-I, relaxin and luteinizing hormone on vascular endothelial growth factor secretion by cultured endometrial stromal cells on different days of early pregnancy in pigs. Reprod. Biol..

[32] Van der Zee E., Everts V., Hoeben K., Beertsen W. (1994). Immunolocalisation of collagenase in rabbit periosteal tissue explants and extraction of the enzyme. The effect of the cytokines IL-1 alpha and EGF. J. Cell Sci..

[33] Szafrańska B., Ziecik A., Okrasa S. (2002). Primary antisera against selected steroids or proteins and secondary antisera against gamma-globulins--an available tool for studies of reproductive processes. Reprod. Biol..

[34] Geisert R.D., Thatcher W.W., Michael Roberts R., Bazer F.W. (1982). Establishment of Pregnancy in the Pig: III. Endometrial Secretory Response to Estradiol Valerate Administered on Day 11 of the Estrous Cycle1,2,3. Biol. Reprod..

[35] Mattson B.A., Overstrom E.W., Albertini D.F. (1990). Transitions in Trophectoderm Cellular Shape and Cytoskeletal Organization in the Elongating Pig Blastocyst. Biol. Reprod..

[36] Keys J.L., King G.J. (1990). Microscopic examination of porcine conceptus-maternal interface between days 10 and 19 of pregnancy. Am. J. Anat..

[37] Bazer F.W., Thatcher W.W. (1977). Theory of maternal recognition of pregnancy in swine based on estrogen controlled endocrine versus exocrine secretion of prostaglandin F2α by the uterine endometrium. Prostaglandins.

[38] McCracken J.A., Custer E.E., Lamsa J.C. (1999). Luteolysis: A Neuroendocrine-Mediated Event. Physiol. Rev..

[39] Krzymowski T., Stefańczyk-Krzymowska S. (2004). The oestrous cycle and early pregnancy—A new concept of local endocrine regulation. Vet. J..

[40] Okrasa S., Franczak A., Zmijewska A., Wojciechowicz B., Dziekonski M., Martyniak M., Kolakowska J., Zglejc K., Kotwica G. (2013). The uterine secretory activity and its physiological changes in the pig. Acta Biol. Cracoviensia. Ser. Zool..

[41] Andronowska A., Postek A., Doboszyńska T. (2006). Epidermal growth factor and epidermal growth factor receptor immunoreactivity in the endothelial cells of the uterine artery and its branches during different stages of the estrous cycle in the pig. Pol. J. Vet. Sci..

[42] Wojciechowicz B., Kotwica G., Kołakowska J., Zglejc K., Martyniak M., Franczak A. (2016). The alterations in endometrial and myometrial transcriptome at the time of maternal recognition of pregnancy in pigs. Agri Gene.

[43] Berg J.M., Tymoczko J.L., Stryer L. (2002). Eukaryotic Transcription and Translation Are Separated in Space and Time. Biochemistry.

[44] Vogel C., Marcotte E.M. (2013). Insights into regulation of protein abundance from proteomics and transcriptomis analyses. Nat. Rev. Genet..

[45] De Sousa Abreu R., Penalva L.O., Marcotte E.M., Vogel C. (2009). Global signatures of protein and mRNA expression levels. Mol. Biosyst..

[46] Gry M., Rimini R., Strömberg S., Asplund A., Pontén F., Uhlén M., Nilsson P. (2009). Correlations between RNA and protein expression profiles in 23 human cell lines. BMC Genomics.

[47] Ashworth M.D., Ross J.W., Stein D.R., Allen D.T., Spicer L.J., Geisert R.D. (2005). Endocrine disruption of uterine insulin-like growth factor expression in the pregnant gilt. Reproduction.

[48] Payne A.H., Hales D.B. (2004). Overview of steroidogenic enzymes in the pathway from cholesterol to active steroid hormones. Endocr. Rev..

[49] Makieva S., Saunders P.T.K., Norman J.E. (2014). Androgens in pregnancy: Roles in parturition. Hum. Reprod. Update.

[50] Nilsson S., Mäkelä S., Treuter E., Tujague M., Thomsen J., Andersson G., Enmark E., Pettersson K., Warner M., Gustafsson J.Å. (2001). Mechanisms of estrogen action. Physiol. Rev..

[51] Franczak A., Wojciechowicz B., Kolakowska J., Zglejc K., Kotwica G. (2014). Transcriptomic analysis of the myometrium during peri-implantation period and luteolysis–the study on the pig model. Funct. Integr. Genomics.

[52] Franczak A., Wojciechowicz B., Kotwica G. (2013). Transcriptomic analysis of the porcine endometrium during early pregnancy and the estrous cycle. Reprod. Biol..

[53] Kiewisz J., Krawczynski K., Lisowski P., Blitek A., Zwierzchowski L., Ziecik A.J., Kaczmarek M.M. (2014). Global gene expression profiling of porcine endometria on Days 12 and 16 of the estrous cycle and pregnancy. Theriogenology.

[54] Simpson E.R., Clyne C., Speed C., Rubin G., Bulun S. (2001). Tissue-specific estrogen biosynthesis and metabolism. Ann. N. Y. Acad. Sci..

[55] Wojciechowicz B., Kotwica G., Zglejc K., Waszkiewicz E., Franczak A. (2017). Expression of 17β-hydroxysteroid dehydrogenase and the effects of LH, FSH and prolactin on oestrone and 17β-oestradiol secretion in the endometrium of pigs during early pregnancy and the oestrous cycle. Reprod. Fertil. Dev..

[56] Ziecik A.J., Przygrodzka E., Jalali B.M., Kaczmarek M.M. (2018). Regulation of the porcine corpus luteum during pregnancy. Reproduction.

[57] Lee C.Y., Green M.L., Simmen R.C.M., Simmen F.A. (1998). Proteolysis of insulin-like growth factor-binding proteins (IGFBPs) within the pig uterine lumen associated with peri-implantation conceptus development. J. Reprod. Fertil..

[58] Simmen F.A., Simmen R.C.M., Geisert R.D., Botte F.M., Bazer F.W., Terqui M. (1992). Differential expression, during the estrous cycle and pre- and postimplantation conceptus development, of messenger ribonucleic acids encoding components of the pig uterine insulin-like growth factor system. Endocrinology.

